# Evaluation of antioxidant deficit in schizophrenia

**DOI:** 10.4103/0019-5545.39753

**Published:** 2008

**Authors:** Gora Dadheech, Sandhya Mishra, Shiv Gautam, Praveen Sharma

**Affiliations:** Department of Biochemistry, SMS Medical College and Hospital, Jaipur (Rajasthan), India; 1 Department of Psychiatry, SMS Medical College and Hospital, Jaipur, (Rajasthan), India

**Keywords:** Antioxidant enzymes, GSHPx, MDA, oxidative stress, schizophrenia, SOD

## Abstract

**Aim:**

Oxidative stress is a state in which there is disequilibrium between pro-oxidant processes and the antioxidant defense system in favor of the former and occurs as a consequence of increased production of free radicals or when the antioxidant defense system is inefficient or a combination of both events. A disturbance in the antioxidant defense system, including antioxidant enzymes superoxide dismutase (SOD) and glutathione peroxidase (GSHPx), due to free radical-induced oxidative injury has also been implicated in various neuropsychiatric disorders. Hence the role of these antioxidant enzymes and the changes in their level in blood and correlation with oxidative stress and the overall mechanism of defense were studied in a common psychiatric illness, schizophrenia.

**Materials and Methods:**

Fifty subjects of either sex ranging in age from 18 to 60 years, divided into two age groups (< 40 years and >40 years), diagnosed for schizophrenia; and 50 age- and sex-matched normal subjects as controls were included in the study. Blood samples were collected for the determination of malondialdehyde (MDA), superoxide dismutase (SOD), glutathione peroxidase (GSHPx), and reduced glutathione (GSH).

**Results:**

Significantly lower levels of the two antioxidant enzymes were found in schizophrenics compared to normal controls, with an increased oxidative stress as indicated by high blood MDA levels. The condition worsened with advancing age, smoking, among literate masses, and in chronic schizophrenics; whereas gender did not show any effect.

**Conclusion:**

It can be concluded that an imbalance in the antioxidant defense system, along with enzymatic antioxidants, occurs in schizophrenia due to the persistent oxidative stress. Modern life style perhaps also contributes to the condition.

## INTRODUCTION

Free radicals are species normally produced during cellular metabolism in aerobic cells, containing one or more unpaired electrons, highly reactive and can combine with a great variety of biomolecules, changing their physicochemical characteristics.[1] The brain tissues contain large amounts of polyunsaturated fatty acids and catecholamines, which are thought to be target molecules for free radical-induced peroxidation and neural cell damage.[2,3] One of the end products of this self-perpetuating lipid peroxidation reaction includes the cytotoxic aldehyde; and malondialdehyde (MDA), which is considered a specific and sensitive measure of lipid auto-oxidation. In conditions of increased production or decreased scavenging of free radicals, an imbalance towards a pro-oxidant state is formed, which may also assume fundamental importance in the pathogenesis of acute or chronic brain diseases[4] like Parkinson's, tardive dyskinesia, schizophrenia.[5,6] Schizophrenia is a common psychiatric disorder, marked by gross distortion from reality; disturbances in thinking, feeling, and behavior[7]; and it is believed that increased oxidative stress[8] may be relevant to the pathophysiology of schizophrenia.[9]

The potential toxicity of free radicals is counteracted by a number of cytoprotective enzymes and antioxidants that limit the damage.[10,11] This protective mechanism functions cooperatively in the form of a cascade in which the various cytoprotective enzymes such as superoxide dismutase (SOD) and glutathione peroxidase (GSHPx); and nutrient, as well as endogenous, antioxidants act in combination.[12,13] In our previous studies, it has been found that the levels of nutrient antioxidants ascorbic acid[14] and α-tocopherol[15] decline as a result of the increasing oxidative stress in schizophrenia. Thus, the present study examines oxidative stress and the activities of antioxidant enzymes SOD and GSHPx, GSH; and the overall mechanism that is disturbed due to the growing oxidative stress. The effects of age, gender, nature of illness, life-style stresses, and smoking on the activities of antioxidants in schizophrenics have also been observed.

## MATERIALS AND METHODS

The study was performed on 50 schizophrenic patients of either sex (39 males and 11 females) ranging in age from 18 to 60 years and divided into two groups: young subjects (< 40 years, *n* = 25) and elderly subjects (>40 years, *n* = 25). All the subjects were thoroughly screened and diagnosed for schizophrenia at the OPD of Psychiatric Center, SMS Medical College, by one of the authors using the ICD-10 diagnosis criterion. A complete clinical and personal history of the subjects was recorded and consent to participate in the study was taken. Subjects with education up to 10^th^ standard were considered literates. The smokers were taking 10 or more cigarettes per day, and nonsmokers were absolutely free from the habit [Table 1]. Psychological evaluation of each subject was done using Positive and Negative Syndrome Scale (PANSS). Subjects were selected using the following exclusion criterion:

**Table 1:** Baseline characteristics of control and schizophrenic subjects

Characteristics	Control (*n* = 50)	Schizophrenics (*n* = 50)
Age (years)	18-60	18-60
<40 (*n*)	28	25
>40 (*n*)	22	25
Gender		
male (*n*)	35	39
female (*n*)	15	11
Education		
literate (*n*)	40	32
illiterate (*n*)	10	18
Smokers (*n*)	18	23
Non-smokers (*n*)	31	27
Passive smokers (*n*)	1	0
Acute stage (*n*)	-	40
Chronic stage (*n*)	-	10

- Pregnant and lactating females
- Symptoms of any other illness
- History of receiving ECT in the last one year
- Any substance dependence for the last one month
- History or present symptoms of any other psychiatric disorder

These were compared with 50 age- and sex-matched normal healthy individuals belonging to the general population and marked as controls. Whole blood samples were drawn from the anticubital vein using aseptic technique. RBCs were separated for estimating malondialdehyde (MDA), an indicator of oxidative stress,[16] measuring it as a secondary fragmentation product of PUFA peroxide, a thiobarbituric acid-reactive substance (TBARS) that gives a pink color complex with thiobarbituric acid (TBA). It was read on a spectrophotometer at 535 nm wavelength. Hemolysate (1:20) was prepared to estimate SOD and GSHPx levels. SOD activity was measured by Randox kit method, using the ability to inhibit the generation of superoxide radicals formed by xanthine and xanthine oxidase activity and reading the degree of inhibition at 505 nm. GSHPx was assayed by measuring the rate of reduction of oxidized glutathione at 340 nm.[17] Reduced glutathione was assayed by reading the reduction of DTNB, forming yellow anion at 412 nm.[18]

### Statistical analysis

Results were statistically explained by Students t-test, and correlation coefficient (r) was done between SOD, GSHPx, GSH, and MDA.

## RESULTS

The results of this study indicate an impaired activity of major intracellular antioxidant enzymes SOD and GSHPx due to increased oxidative stress inherent in schizophrenia. The level of MDA, an indicator of oxidative stress and an end product of lipid peroxidation, measured as thiobarbituric acid-reactive substance (TBARS), was significantly (*P* < 0.001) higher in schizophrenics, indicating increase in level of oxidative stress due to schizophrenia.[9] The SOD and GSHPx activities in red cells, which act as sinks for O_2_^.-^ and H_2_ O_2_ produced in plasma, decreased significantly (*P* < 0.001) in schizophrenics as compared to controls [Table 2]. A significant (*P* < 0.001) reduction in the level of reduced glutathione with increasing oxidative stress [Table 2] was also observed.

**Table 2:** Oxidative stress and antioxidants in controls and schizophrenics

Characteristics	Control (*n* = 50)	Schizophrenics (*n* = 50)	Significance
MDA (nmol/gmHb)	0.047 ± 0.005 (0.040-0.062)	0.061 ± 0.010* (0.040-0.093)	*P* < 0.001
SOD (U/gmHb)	790.3 ± 64.2 (640.2-892.8)	479.8 ± 70.1* (391.8-605.3)	*P* < 0.001
GSHPx (U/gmHb)	11.82 ± 2.59 (8.00-16.64)	5.60 ± 1.41* (3.55-8.61)	*P* < 0.001
Reduced GSH (mg/dl)	41.05 ± 5.89 (31.30-50.00)	35.40 ± 3.92* (29.40-40.00)	*P* < 0.001

Data in parentheses represent range; Values are mean ± SD

An imbalance between oxidative stress and antioxidants in favor of oxidative stress was observed in schizophrenics in the present study [Table 3], showing that lipid peroxidation is fundamental in the aging process.[22] However, no significant difference could be observed in oxidative stress and antioxidants when compared between males and females [Table 4].

**Table 3:** Effect of age on oxidative stress and antioxidant enzyme levels in controls and schizophrenics

Age range	Controls (*n* = 50)		Schizophrenics (*n* = 50)	
		
(*n* = 25)	<40 yrs. (*n* = 28)	>40 yrs. (*n* = 22)	<40 yrs. (*n* = 25)	>40yrs.
MDA (nmol/gmHb)	0.044 ± 0.003	0.051 ± 0.005*	0.053 ± 0.006	0.073 ± 0.010*
SOD (U/gmHb)	821.74 ± 61.43	750.35 ± 42.33*	550.40 ± 33.17	439.06 ± 16.27*
GSHPx (U/gmHb)	11.82 ± 2.70	11.83 ± 2.49	5.89 ± 0.03	4.01 ± 0.22*
GSH (mg/dl)	44.81 ± 4.31	36.26 ± 3.76*	39.02 ± 0.67	30.01 ± 1.91*

Statistical comparison was done between control group (<40 years and >40 years) and schizophrenic group (<40 years and >40 years);

* *P* < 0.001; values are mean ± SD

**Table 4:** Effect of gender on oxidative stress and antioxidant enzyme levels in controls and schizophrenics

Gender	Controls (*n* = 50)		Schizophrenics (*n* = 50)	
		
	Male (*n* = 35)	Female (*n* = 15)	Male (*n* = 39)	Female (*n* = 11)
MDA (nmol/gmHb)	0.047 ± 0.006	0.047 ± 0.004	0.061 ± 0.019	0.060 ± 0.022
SOD (U/gmHb)	793.47 ± 72.42	783.00 ± 40.38	499.01 ± 76.80	495.06 ± 40.00
GSHPx (U/gmHb)	11.93 ± 2.62	11.58 ± 2.58	5.60 ± 1.01	5.42 ± 0.04
GSH (mg/dl)	41.84 ± 6.02	39.19 ± 5.28	36.00 ± 4.19	35.60 ± 3.02

Statistical comparison was done between control group (male and female) and schizophrenic group (male and female). Values are mean ± SD

The study also examined if there is any contribution of risk factors related to habits or if these factors are associated with oxidative stress. Literates both in controls and schizophrenics were found to be at greater risk as they had significantly higher MDA level as compared to illiterates [Tables 5,6]. A strong negative correlation was found between MDA, a measure of oxidative stress, and SOD (r = -0.88); between MDA and GSHPx (r = -0.87); and between MDA and GSH (r = -0.81) [Table 7].

**Table 5:** Distribution of baseline characteristics and antioxidant levels in quartiles with respect to oxidative stress in control subjects

Quartile	1 (*n* = 14) – 0.044	2 (*n* = 13) – 0.046	3 (*n* = 10) – 0.049	4 (*n* = 13) – 0.062	*P*
Age (years)	34.00 ± 6.36	35.61 ± 7.43	45.60 ± 8.97	43.53 ± 9.42	0.03
Gender				
male (*n*)	13	7	7	8	
female (*n*)	1	6	3	5	
Education				
literate (*n*)	8	10	10	12	
illiterate (*n*)	6	3	0	1	
Smokers (*n*)	1	1	6	10	
Non-smokers (*n*)	12	12	4	3	
Passive smokers (*n*)	1	0	0	0	
MDA (nmol/gmHb)	0.042 ± 0.001	0.045 ± 0.001	0.048 ± 0.001	0.056 ± 0.004	<0.01
SOD (U/gmHb)	862.0 ± 31.90 (793.6-886.5)	793.7 ± 54.14 (641.2-841.7)	770.1 ± 23.74 (712.2-793.6)	730.7 ± 41.21 (640.2-757.5)	<0.01
GSHPx (U/gmHb)	11.72 ± 2.57 (8.00-16.64)	11.22 ± 2.44 (8.00-14.50)	11.80 ± 2.97 (8.72-16.64)	12.56 ± 2.57 (8.72-16.64)	0.05
Reduced GSH (mg/dl)	46.58 ± 2.18 (43.10-50.00)	45.01 ± 2.86 (41.10-50.00)	42.10 ± 1.68 (36.00-41.00)	39.39 ± 1.62 (31.30-36.00)	<0.01

Data in parentheses represent range; Values are mean ± SD; *P* test of linear trend across quartiles

**Table 6:** Distribution of baseline characteristics and antioxidant levels in quartiles with respect to oxidative stress in schizophrenics

Quartile	1 (*n* = 14) – 0.054	2 (*n* = 11) – 0.067	3 (*n* = 15) – 0.090	4 (*n* = 10) – 0.098	*P*
Age (years)	34.02 ± 4.01	33.01 ± 8.08	44.29 ± 7.01	42.01 ± 6.42	0.03
Gender				
male (*n*)	14	6	11	8	
female (*n*)	0	5	4	2	
Education				
literate (*n*)	5	7	11	10	
illiterate (*n*)	9	4	4	0	
Smokers (*n*)	2	1	9	10	
Non-smokers (*n*)	12	10	6	0	
Passive smokers (*n*)	0	0	0	0	
Acute stage (*n*)	12	10	10	8	
Chronic stage (*n*)	2	1	5	2	
MDA (nmol/gmHb)	0.049 ± 0.001	0.062 ± 0.004	0.077 ± 0.003	0.092 ± 0.003	<0.01
SOD (U/gmHb)	578.8 ± 27.09 (534.1-620.3)	534.9 ± 48.04 (478.9-617.2)	450.2 ± 30.02 (400.6-520.8)	422.6 ± 18.21 (384.0-458.7)	<0.01
GSHPx (U/gmHb)	7.12 ± 0.70 (6.40-8.61)	6.29 ± 0.81 (4.57-7.46)	4.03 ± 0.13 (3.69-5.71)	4.10 ± 0.01 (3.55-4.80)	<0.01
Reduced GSH (mg/dl)	39.77 ± 0.37 (38.80-40.00)	37.60 ± 2.35 (30.00-38.80)	31.00 ± 3.05 (29.40-37.20)	29.02 ± 2.05 (29.40-35.20)	<0.01

Data in parentheses represent range; Values are mean ± SD; *P* test of linear trend across quartiles

**Table 7:** Correlation	between oxidative stress and antioxidant levels in schizophrenics

Groups	Correlation coefficient (r)
MDA – SOD	-0.88
MDA – GSHPx	-0.87
MDA – GSH	-0.81

## DISCUSSION

The increase in oxidative stress in the study seems to be a result of the toxic effects of free radicals, as they cause membrane defects, oxidation of the lipid membranes and also increase the rate of catecholamine oxidative metabolism,[5] all of which may play an important role in the pathophysiology of schizophrenia.[19] Further, the condition worsens due to advancing age, smoking, and in literates with a modern stressful life style and chronic stage of illness.

The modern stressful living, especially in the literate subjects, and self-pollution in the form of smoking have worsened the situation, thus contributing to oxidative stress. This can be attributed to the disturbance in the overall mechanism of generation of free radicals and their consequent neutralization due to oxidative stress in schizophrenia, where SOD is utilized for neutralizing the free radical superoxide ion (O_2_^.-^) to H_2_ O_2_ and oxygen, and GSHPx reduces this H_2_ O_2_ to water.[20] Schizophrenia also seems to disturb the glutathione homeostasis, which is also one of the factors responsible for weakening the antioxidant defense provided by enzymatic antioxidants.[3,21]

Age seems to have a definite impact on lipid peroxidation in the presence of antioxidant deficit. This imbalance in the antioxidant enzyme levels may be attributed as a resistance against the developing stress because the antioxidant enzymes are the first line of defense.[23]

The disturbance of the oxidant-antioxidant equilibrium now becomes quite evident in schizophrenia owing to the abnormal activity of the critical enzymes SOD and GSHPx. Depleted antioxidant status due to increased utilization with increasing oxidative stress has been reported.[9,23,24] However, few studies have advocated strengthening of antioxidant status in schizophrenia so as to counteract oxidative stress.[25–27] This difference in observation may probably be attributed to varied clinical symptoms or therapeutic features or duration of the illness. In the present study, all the participant schizophrenics had never taken any treatment earlier and had come for consultation for the first time.

Thus, a disturbance in the activities of antioxidant enzymes occurs in schizophrenia due to the increased oxidative stress, which further intensifies with the aging process and chronic stage of the illness. The gift of modern world, a stressful life; and a polluted environment also take their toll and deteriorate the condition of the schizophrenics. This also emphasizes the importance of nutrient antioxidant supplementation to support the entire antioxidant defense system.
